# Interactive wearable systems for upper body rehabilitation: a systematic review

**DOI:** 10.1186/s12984-017-0229-y

**Published:** 2017-03-11

**Authors:** Qi Wang, Panos Markopoulos, Bin Yu, Wei Chen, Annick Timmermans

**Affiliations:** 1 Department of Industrial Design, Eindhoven Technology University, Eindhoven, The Netherlands; 2 Center for Intelligent Medical Electronics, Department of Electronic Engineering, Fudan University, Shanghai, China; 3 Shanghai Key Laboratory of Medical Imaging Computing and Computer Assisted Intervention, Shanghai, China; 4 BIOMED REVAL Rehabilitatio Research Institute, Faculty of Medicine and Life Sciences, Hasselt University, Diepenbeek, Belgium

**Keywords:** Wearable technology, Rehabilitation, Interactive feedback, Upper body, Posture monitoring, Motion sensing

## Abstract

**Background:**

The development of interactive rehabilitation technologies which rely on wearable-sensing for upper body rehabilitation is attracting increasing research interest. This paper reviews related research with the aim: 1) To inventory and classify interactive wearable systems for movement and posture monitoring during upper body rehabilitation, regarding the sensing technology, system measurements and feedback conditions; 2) To gauge the wearability of the wearable systems; 3) To inventory the availability of clinical evidence supporting the effectiveness of related technologies.

**Method:**

A systematic literature search was conducted in the following search engines: PubMed, ACM, Scopus and IEEE (January 2010–April 2016).

**Results:**

Forty-five papers were included and discussed in a new cuboid taxonomy which consists of 3 dimensions: sensing technology, feedback modalities and system measurements. Wearable sensor systems were developed for persons in: 1) Neuro-rehabilitation: stroke (*n* = 21), spinal cord injury (*n* = 1), cerebral palsy (*n* = 2), Alzheimer (*n* = 1); 2) Musculoskeletal impairment: ligament rehabilitation (*n* = 1), arthritis (*n* = 1), frozen shoulder (*n* = 1), bones trauma (*n* = 1); 3) Others: chronic pulmonary obstructive disease (*n* = 1), chronic pain rehabilitation (*n* = 1) and other general rehabilitation (*n* = 14). Accelerometers and inertial measurement units (IMU) are the most frequently used technologies (84% of the papers). They are mostly used in multiple sensor configurations to measure upper limb kinematics and/or trunk posture. Sensors are placed mostly on the trunk, upper arm, the forearm, the wrist, and the finger. Typically sensors are attachable rather than embedded in wearable devices and garments; although studies that embed and integrate sensors are increasing in the last 4 years. 16 studies applied knowledge of result (KR) feedback, 14 studies applied knowledge of performance (KP) feedback and 15 studies applied both in various modalities. 16 studies have conducted their evaluation with patients and reported usability tests, while only three of them conducted clinical trials including one randomized clinical trial.

**Conclusions:**

This review has shown that wearable systems are used mostly for the monitoring and provision of feedback on posture and upper extremity movements in stroke rehabilitation. The results indicated that accelerometers and IMUs are the most frequently used sensors, in most cases attached to the body through ad hoc contraptions for the purpose of improving range of motion and movement performance during upper body rehabilitation. Systems featuring sensors embedded in wearable appliances or garments are only beginning to emerge. Similarly, clinical evaluations are scarce and are further needed to provide evidence on effectiveness and pave the path towards implementation in clinical settings.

## Background

In musculoskeletal disorders, such as disorders of the neck-shoulder complex or osteoporosis, and in neurological disorders such as stroke, the integration of posture awareness of the upper trunk and shoulder complex as a stable basis for upper limb movement is an essential component of rehabilitation [1–3]. Therefore feedback on the posture of the trunk and shoulder complex and feedback on upper limb movement may be supportive of motor learning [4]. Although the pathological mechanisms of posture deviation during static conditions (standing, sitting) or during movement performance (upper limb activities, posture during gait) are quite different across the above mentioned patient populations the corresponding therapeutic approaches share an emphasis on increasing patient awareness of correct posture and movement patterns and the provision of corrective feedback during functional task execution. In all of the above patients, intrinsic feedback mechanisms that inform the patient (e.g. proprioceptive cues) are impaired [5–7] and extrinsic feedback is advocated to relearn correct joint positions/posture during movement. Traditionally extrinsic feedback is provided by a therapist, so this way of learning is very time consuming and difficult to carry out independently, e.g. during home exercises. Suitable rehabilitation technologies can potentially play an instrumental role in extending training opportunities and improving training quality.

Posture monitoring and correction technologies providing accurate, and reliable feedback, may support current rehabilitation activities [8, 9]. Ideally feedback is given continuously for users with low proficiency levels, and with fading frequency schedules for more advanced users [8]. In broad terms, there are five kinds of monitoring methods available: 1) traditional mechanical systems (e.g. goniometer); 2) optical motion recognition technologies [10]; 3) marker-less off body tracking systems like depth camera-based movement detection systems (e.g. Microsoft Kinect [11, 12]); 4) Robot-based solutions [13, 14]; 5) wearable sensor-based systems [4]. Recently, the miniaturization of devices, the evolution of sensing and body area network technologies [15, 16] has triggered the increasing influence of wearable rehabilitation technology, offering advantages over traditional rehabilitation services [17, 18], such as: low cost, flexible application, remote monitoring, comfort. Wearable sensing systems open up the possibility of independent training, the provision of feedback to the end-user as an active monitoring system, or even tele-rehabilitation.

A great number of wearable posture/motion monitoring systems for rehabilitation have been reported in literature in recent years, though very few have been used in clinical studies. Some studies introduce innovative wearable sensing technologies, e.g. Kortier et al. [19] developed a hand kinematics assessment glove based on attaching a flexible PCB structure on the finger that contains inertial and magnetic sensors. Tormene et al. [20] proposed monitoring trunk movements by applying a wearable conductive elastomer strain sensor. Studies like this are primarily concerned with demonstrating the accuracy and reliability of the technology they introduce. Another body of research concerns evaluations of existing rehabilitation technologies in terms of their validity. For example, Uswatte et al. [21] conducted a validation study of accelerometry for monitoring arm activity of stroke patients. Bailey et al. [22] proposed a study on a accelerometry-based methodology for the assessment of bilateral upper extremity activity. Lemmens et al. [23] report a proof of principle for recognizing complex upper extremity activities using body worn sensors.

There are a few examples of a literature that grows fast. The need arises to classify related works and identify promising trends or open challenges in order to guide future research. To address this need, there have been several reviews of research on wearable systems for rehabilitation, which take quite diverse perspectives on this vibrant field. An early review by Patel et al. [16] takes a very broad perspective that covers health and wellness, rehabilitation and even prevention, reviewing wearable and ambient technologies. Hadjidj et al. [24], provide an non-systematic review of literature on wireless sensor technologies focusing on technical requirements. Some studies focus on physical activity monitoring [25, 26] a technology domain that has had substantial growth and impact, but which is not specific to rehabilitation. Allet et al. [26] review wearable systems for monitoring mobility related activities in chronic diseases; this review covered mostly systems measuring general physical activity and found no works reaching the stage of clinical testing. Some studies provide an in-depth overview of movement measurement and analysis [27–29] technologies, though these are not necessarily integrated in rehabilitation systems and are usually still at the stage of proof of principle for a measurement technique. Vargas et al. [30] reviewed inertial sensors applied in human motion analysis, and concluded that inertial sensors can offer a task-specific accurate and reliable method for human motion studies. A couple of recent surveys [31, 32] have reviewed e-textile technologies applied in rehabilitation, though one of their main conclusions was to identify the distance separating the requirements for applying textiles to rehabilitation from the current state of the art. Also, they identify that the potential of providing feedback to patients based on textile sensing remains largely unexplored. Some studies concentrated specifically about how feedback influences therapy outcome [33–35], however the systems involved are not only wearable systems and all these reviews date 6 years or longer. Wang et al. [9] reviewed wearable posture monitoring technology studies from 2008 to 2013 for upper-extremity rehabilitation, yet unlike the present article, no systematic comparisons based on technology, system usability, feedback and clinical maturity were provided. In line with Fleury et al. [32] they found that only a few studies report the integration of wearable sensing in complete systems supporting feedback to patients, and very few of those have been tested by users with attention to the usability and wearability. Given the limited nature of that survey, such a conclusion was tentative calling for a systematic survey to gauge the state of the art in upper body rehabilitation technologies that integrate wearable sensors. The focus of the present survey is different regarding to the sensor type and placement, and rehabilitation objective. The present article contributes a different perspective to these surveys by critically reviewing and comparing systems comprising of feedback to support upper body rehabilitation with regard to their functionality and usability. In this review we focus on interactive wearable systems that provide feedback to end-users for rehabilitation. In addition, in order to review the latest and most innovative technological solutions that shed a light on the state of the art wearable solutions for rehabilitation, only articles published later than 2010 are considered.

The translation from a technical tool towards a clinically usable system is not straightforward. Prerequisites for therapists and patients to use technology supported rehabilitation systems are the easy-to-use character of the system, its added value to their habitual rehabilitation programs and its credibility. Besides, it is of major importance to design the system feedback as this positively influences motivation and self-efficacy [8]. Advanced technologies provide increasing possible forms of feedback and a growing number of studies used interactive wearable systems to motivate patients in the intensive and repetitive training.

As such, the purpose of this review is to provide an overview of interactive wearable systems for upper body rehabilitation. In particular, we aim to classify from the following aspects:

1. To inventory and classify interactive wearable systems for movement and posture monitoring during upper body rehabilitation, regarding the sensing technology, system measurements and feedback conditions;
2. To gauge the wearability of the wearable systems;
3. To inventory the availability of clinical evidence supporting the effectiveness of related technologies.

## Method

### Literature search strategy

A literature search was conducted in the following four databases: PubMed, IEEE Xplore, ACM and Scopus. Papers addressing the following aspects were selected: rehabilitation, upper body, posture/motion monitoring, and wearable systems. MeSh (Medical Subject Heading) terms or Title/Abstract keywords and their synonyms and spelling variations were used in several combinations and modified for every database. Articles published from January 2010 to April 2016 were reviewed. The general search strategy including the used search terms are listed in Table 1 This search includes refereed journal papers and peer reviewed articles published in conference proceedings. Only English articles are included.

**Table 1:** Literature search strategy

Rehabilitation	“rehabilitation” OR “telerehabilitation” OR “motor activity” OR “physical therapy” OR “telemedicine” OR telemetry OR “motor learning”
	AND
Upper body	“upper body” OR “upper extremity” OR “spine” OR “back” OR “arm hand” OR “shoulder” OR “elbow” OR “wrist” OR “joint”
	AND
Posture/movement monitoring	(“monitor” OR “motion” OR “posture” OR “sensing” NOT “walking”) OR (“acceleromet*” OR “inertial sensor” OR “ sensor system” OR “sensor network” OR gyroscope OR MEMS OR IMU)
	AND
Wearable systems	wearable OR garment OR textiles OR wireless OR mobile OR “smart phone”

### Study selection process

**Fig. 1:**
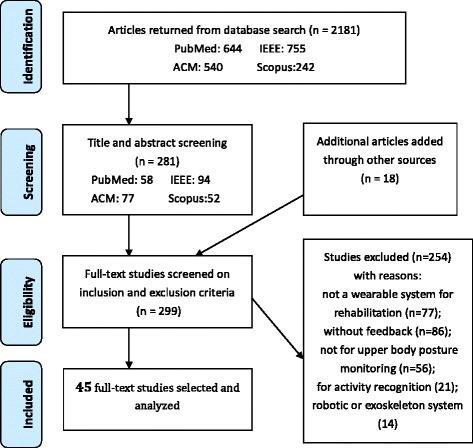
Prisma [36] flowchart of the results from the literature search

The article selection process consisted of following steps using the PRISMA [36] guidelines (see Fig. 1): 1) A computerized search strategy was performed for the period January 2010 until April 2016; 2) After removal of duplicates, two independent reviewers (QW and BY) screened titles and abstracts of the remaining articles; 3) The same 2 independent reviewers read the full texts and selected articles based on the inclusion/exclusion criteria. In cases where a journal paper covered the contents reported in the earlier conference publications, the journal paper was preferred over the conference paper. In cases where the overlap was only partial, multiple publications were used as sources, but only counted as one in our statistics and table entries. The consensus rates were 90.5 and 81% respectively during the first and second review rounds; disagreement was resolved by discussing reasons for exclusion. When authors had published several studies on same research initiative, only the most recent studies were retained. In cases of disagreement between the two reviewers, a third reviewer (WC/AT) decided whether the article should be included or not.

- Inclusion criteria were:
  - a) The articles concern a wearable system.
  - b) The system is intended for rehabilitation purposes (in home and community settings).
  - c) The study includes upper body training (upper extremity, neck, spine).
  - d) The system described is a movement tracking or posture monitoring system
  - e) The wearable systems provide feedback to the end users of their training results or performance
  - g) Articles were published in the last 6 years
  - h) Articles were written in English
- Exclusion criteria:
  - a) Prosthetics, coaching and information/educational systems
  - b) Activity recognition systems
  - c) Robotic system or exoskeleton
  - d) The study sticks adhesive sensors to human skin directly
  - e) Reviews
  - f) Books

### Data extraction process

Two researchers (QW and BY) extracted data independently according to a predetermined template. The extracted data included the technology used, the sensor placement, the feedback, validation test level, the wearability of the system, and its purpose (patient category, posture or trunk rehabilitation). As for feedback, the researchers classified feedback according to the feedback modality (knowledge of results feedback/knowledge of performance feedback, concurrent/terminal, vibrotactile/auditory/visual). With regard to the level of validation, it was noted whether the paper reports a technical performance evaluation, an empirical usability test, or a clinical trial to assess the effectiveness of the technology. In addition, this review follows the taxonomy of (WSN) for clinical rehabilitation applications proposed by Hadjidj et al. [13] in 2013.

## Results

### Database search and paper lists

An overview of the results in the different stages of the article selection process is shown in Fig. 1. From the 2181 articles that were identified with the search strategies, 45 papers are included in this review after the selection process. The primary features of the surveyed systems are summarized and compared in Table 2.

**Table 2:** Summary of the paper lists and features

Reference	Technology (n)	Placement	Feedback			Evaluation		Wearability	Measurement &Rehabilitation Purpose	Target population
Lee et al. 2010; [37]	Accel (*n* = 2)	Upper arm, Forearm	KP	Concurrent; Kinematic rendering of upper extremity.	VR	Tech.	Accuracy experiment on rotary stage and comparison experiment with goniometer (*n* = 1)	Strapped with Velcro; Attachable;	ROM; Improve active joint range of motion;	General rehabilitation (arm)
Kapur et al. 2010; [38]	Magnetometer (*n* = 3)	Wrist, Elbow, Clavicle	KP, KR	Concurrent; Kinematic rendering of upper extremity, Vibration for movement direction; Prescriptive KP FB;	VR; Vibrotactile FB	None	N/A	Full-body Suit; Embedded, normal wire;	Body Segment Posture; Improve movement performance;	Stroke
Luo et al. 2010; [39]	OLE (6), IMU (*n* = 2)	Upper arm, Elbow, Wrist, Finger	KR	Concurrent; VR game based on Kinematic rendering of upper extremity and hand, Sound FB.	VR (game); Auditory FB	Tech.	Reliability Test (*n* = 5);	Arm Suit & Glove; Attachable;	Body Segment Posture; Improve movement performance;	Stroke
Wang et al. 2010; [40]	Accel (up to 8),	Upper arm, Elbow, Wrist	KP, KR	Concurrent; Movement curve of each sensor node, Video of patient’s training.	Visual FB (PC, PDA)	Usab.	Pre-clinical evaluation trial on patients (*n* = 2)	Strapped with Fabric bands; Attachable;	Speed & Body Segment Posture; Improve movement performance;	Ligament injury, Hemiplegic rehabilitation
Wai et al. 2010; [41]	IRIS mote (accel & gyro)	Upper arm Forearm	KP, KR	Concurrent; Kinematic rendering of body, Sensor Reading.	Visual FB (PC)	Tech.	Accuracy measurement on subject	Based on straps; Attachable;	Body Segment Posture; Improve movement performance;	General rehabilitation
Dunne et al. 2010; [42]	Accel (*n* = 1)	Chest	KR	Concurrent; Game score based on trunk posture, BW FB on trunk compensatory movement.	Visual FB (Multi-Touch Display, game)	None	N/A	Portable device, Attachable;	Body Segment Posture; Improve posture and movement performance;	Cerebral palsy children
Timmermans et al. 2010; [4]	IMU (*n* = 3)	Chest, Upper arm, Forearm	KP, KR	Concurrent & Terminal; Gauge represents real-time joint angle, Avatar animation of upper body, Movement parameters score, etc.;	Visual FB (PC)	Usab. Clini.	Clinical evaluation (*n* =9)	Garment on torso & Strap on arm; Attachable;	ROM & Body Segment Posture; Improve active joint range of motion; Improve performance of ADL skills.	Stroke
Harms et al. 2010; [43]	Accel (*n* = 2)	Forearm, Upper arm	KP	Concurrent; Avatar animation of upper body;	Visual FB	Tech.	Accuracy test on normal people (*n* = 5)	Embedded into a wearable garment; Embedded;	ROM; Improve posture; Improve active joint range of motion;	General Rehabilitation (Shoulder and Elbow)
Markopoulos et al. 2011; [44]	Accel (*n* = 2)	Wrist	KR	Terminal; Summary FB of arm use;	Visual FB (Watch-like device)	Usab.	Preclinical & Usability Test on real patients (*n* = 9)	Watch-like device; Attachable;	Amount of Use; Overcome learned non-use; Improve performance of ADL skills.	Stroke
Moya et al. 2011; [45]	IMU (*n* = 4)	Back, Shoulder, Elbow, Wrist	KP KR	Concurrent; Avatar animation of bodyand arm movement, Sphere animation for trajectory indication, Repetition number, progress bar; Prescriptive;	VR (game)	Usab.	Preclinical & Usability Test (*n* = 23 therapists)	Special garment fixes sensor with Velcro; Attachable;	Body Segment Posture; Improve movement performance;	Stroke
Nguyen et al. 2011; [46]	OLE, Accel	Shoulder, Elbow, Wrist	KP	Concurrent; Kinematic model of upper arm and forearm; Prescriptive KP;	VR	Tech.	Comparison experiments for accuracy andperformance (*n* = 3); Repeatability and Reliability test (n = 5)	Fabric sensor package with Velcro strap; Embedded;	ROM;Improve active joint range of motion;	Stroke
Ambar et al. 2012; [48]	Flex sensor, Accel	Elbow, Forearm, Wrist	KR	Terminal; The number of the arm bent movement;	Visual FB (LCD)	Tech.	1, sensor performance report; 2, Comparison Experiment of FSRs and EMG; 3, ACC measurement activity.	Strapped with Velcro; Attachable;	Amount of Use; Improve performance of ADL skills.	Stroke
Alankus et al. 2012; [49]	Accel (Wii built-in, *n* = 2)	Back, Upper arm	KR	Concurrent; Game result based on both trunk and arm movement; BW FB for trunk and arm compensatory movement;	Screen Visual FB (PC game); Auditory FB	Tech. Usab.	Validation Test on normal people (*n* = 2); Preclinical & Usability Test on patients (*n* = 11)	Harness with straps around the neck, armband; Attachable;	Body Segment Posture; Improve posture and movement performance;	Stroke
Delbressine et al. 2012; [3]	Accel (*n* = 2)	Back, Shoulder	KR	Concurrent; BW FB on trunk or shoulder compensatory movement with vibration;	Visual FB (Touchscreen)	Usab.	Preclinical & Usability Test on patients (*n* = 7)	Vest; Attachable; pocket	Body Segment Posture; Improve posture; Improve performance of ADL;	Stroke
Jeong et al. 2012; [50]	Accel (*n* = 1)	Wrist	KR	Concurrent & Terminal; Speed, count, time of cycling movement;	Visual FB (PC)	Tech.	Accuracy test on normal people (*n* = 7)	Strapped with Velcro; Attachable;	Amount of Use & Speed; Encourage arm hand use; Motivate exercise performance;	General Rehabilitation (arm)
Myllymaa et al. 2012; [51]	Accel (*n* = 1)	Arm	KP	Concurrent; Motion instruction; Prescriptive KP FB;	Vibrotactile FB,	None	N/A	Strapped with band; Attachable;	Amount of Use; Improve muscle strength;	General Rehabilitation (arm)
Ding et al. 2013; [52]	IMU (*n* = 2)	Forearm, Upper arm	KP, KR	Concurrent; Indication of movement direction for upper extremity; BW FB for the reference position; Prescriptive KP FB;	Vibrotactile FB	Tech.	Accuracy test on subjects (*n* = 5)	Strapped with Velcro; Attachable;	Body Segment Posture; Improve posture and movement performance;	Stroke
Saggio et al. 2013; [53]	Flex sensor (*n* = 5)	Finger, Palm	KP	Concurrent; Hand avatar;	Visual FB (PC)	Tech. Usab.	Repeatability and reliability test on normal people (*n* = 6); Usability test on normal people (*n* = 6)	Glove;Attachable;	ROM; Improve active joint range of motion;	General Rehabilitation (hand)
Spina et al. 2013; [54]	IMU (Smartphone built in)	Limb segment	KR KP	Concurrent & Terminal; Teach mode and Train mode; Error notification, repetition number, motion speed; Prescriptive KP;	Visual FB (Smartphone); Auditory FB	Usab.	Usability test on normal people (*n* = 4) and patients (*n* = 7)	Strapped with holster; Attachable;	ROM; Improve active joint range of motion;	COPD (Chronic pulmonary obstructive disease rehabilitation)
Bleser et al. 2013; [55]	IMU (up to 10)	Chest, Upper arm, Forearm, Pelvis	KR KP	Concurrent & Terminal; Movement Error notification and correction FB; Training time, repetition number, progress bar; Descriptive;	Visual FB (Television screen); Auditory FB	Tech. Usab.	Technical evaluation based on therapy ground truth (*n* = 7) Clinical evaluation focusing on usability and acceptance on patients (*n* = 30)	Based on Velcro straps and elastic silicon; Attachable;	ROM & Body Segment Posture; Improve active joint range of motion; Improve movement performance;	General Rehabilitation;
Daponte et al. 2013; [56]	IMU (>2)	Limb segment (Upper arm, Forearm)	KP	Concurrent; Avatar animation of arm movement, Real-time joint angle & ROM value;	VR	Tech.	Function validation test (*n* = 1)	Velcro based Sleevelet units; Embedded;	ROM; Improve active joint range of motion;	General Rehabilitation;
Luster et al. 2013; [57]	Accel (*n* = 2),	Wrist	KR	Concurrent; BW FB on impaired arm use;	Vibrotactile FB,	Usab.	Usability test of vibration Feedback, Stroke patients (*n* = 4)	Wristband; Embedded;	Amount of Use; Overcome learned non-use;	Stroke
Bhomer et al.[58], 2013;	Stretch sensing fabric	Arm, low back	KP	Concurrent; Sound FB matched the arm and low back movement, Movement region indication;	Visual FB (Smartphone); Auditory FB	Usab.	Usability test with therapist and patients	Knitted garment; Integrated;	ROM; Improve active joint range of motion;	Alzheimer
Friedman et al. 2014; [47]	Electrical lead (*n* = 6)	Finger	KR	Concurrent; Game result based on finger pinch and grasp motion;	Screen Visual FB (PC game); Auditory FB	Usab. Randomized Clic.	Usability test with storke patients (*n* = 10) Randomized clinical trial with stroke patients (*n* = 12).	Glove; Integrated;	Body Segment Posture; Improve coordination; Improve performance of ADL skills.	Stroke
Ferreira et al. 2014; [59]	IMU (Smartphone built-in)	Wrist	KR	Concurrent; Game results on arm movement performance;	Visual FB (Smartphone game)	Tech. Usab.	Accuracy measurement on structure, Usability of the system on patients (*n* = 1)	Wristband; Attachable;	Body Segment Posture; Improve movement performance;	Stroke
Fortino et al. 2014; [60]	Accel (*n* = 2)	Upper arm, Elbow	KP, KR	Concurrent & Terminal; Kinematic rendering of limb bending movementperformance, Improvement index data; Descriptive;	Visual FB (Smartphone)	Tech.	Estimation accuracy on normal subject (*n* = 1)	Elastic bracelets; Attachable;	ROM; Improve active joint range of motion;	General Rehabilitation (arm)
Panchanathan et al. 2014; [61]	IMU (n > 1)	Upper arm, Forearm	KP	Concurrent; Vibration instruction for position and speed error;	Vibrotactile FB	Usab.	Usability test withnormal people (*n* = 16)	Sleeve; Embedded; Conductive ribbon	Body Segment Posture & Speed; Improve movement performance;	General Rehabilitation(arm)
Rahman et al. 2014; [62]	LDR sensor	Hand	KR	Terminal; Game score on arm movement;	VR (game)	Tech.	Accuracy and sensitivity test on normal people (*n* = 2)	Fabric Glove; Embedded; Normal wire	Body Segment Posture; Improve movement performance;	Cerebral Palsy
Salim et al. 2014; [63]	Tilt sensor (*n* = 2)	Hand	KP	Concurrent; Indication of arm movement direction;	Visual FB (Smartphone)	Tech.	Pilot experiment (*n* = 1)	Fabric Glove; Embedded; Normal wire	Body Segment Posture; Improve movement performance;	Stroke
Friedman et al. 2014; [64]	Magnetometer (*n* = 2), Accel (*n* = 1)	Wrist, finger	KP	Concurrent; Amount of use of wrist and finger;	Visual FB (Tablet)	Tech.	Accuracy test on normal peoplev (*n* = 7)	Watch-like device, ring; Attachable;	Amount of Use; Encourage arm hand use; Motivate exercise performance;	General Rehabilitation (hand)
Parker et al. 2014; [65]	IMU (*n* = 3)	Chest, Upper arm, Wrist	KP, KR	Concurrent & Terminal; Characteristics and results of performance by avatar presentation and charts; Prescriptive;	Visual FB (PC)	Usab.	Home-based on usability test on real patients (*n* = 5)	Garment on torso & Strap on arm; Attachable;	ROM; Improve active joint range of motion; Improve performance of ADL skills;	Stroke
Halic et al. 2014; [66]	Flex sensor (*n* = 5),	Finger	KP, KR	Concurrent; Avatar presentation, Game score;	Visual Game (Smartphone)	Usab.	Usability test with normal people (*n* = 46)	Glove; Attachable;	Body Segment Posture; Improve movement performance;	General Rehabilitation (hand and wrist)
Tsekleves et al. 2014; [67]	IMU (Wii plus built-in, *n* = 2)	Upper arm, Forearm	KR, KP	Concurrent & Terminal; Avatar presentation, game score, ROM value;	VR (game)	Tech. Usab. Clic.	Accuracy test on structure; Usability test with stroke patients (*n* = 3); Small Clinical trial (*n* = 1)	Based on strips; Attachable;	Body Segment Posture; Improve active joint range of motion; Improve movement performance;	Stroke
Moreira et al. 2014; [68]	IMU (*n* = 11)	Finger, Palm	KP	3d hand model animation,	VR	Tech.	Accuracy test with structure; Reliability evaluation on normal people (*n* = 1);	Glove; Embedded;	ROM; Improve active joint range of motion;	General Rehabilitation (hand)
O’Flynn et al. 2015; [69]	IMU (*n* = 16)	Finger, Palm	KR,KP	Concurrent & Terminal; Movement error summary; 3d hand model animation, dynamic movement value& analysis;	VR	Tech.	Comparison accuracy test on structure.	N/A	ROM; Improve active joint range of motion;	Arthritis Rehabiliation
Hermanis et al. 2015; [70]	IMU (up to 200)	Back	KP	Concurrent; 3D rendering surface showing the current posture of back or limb;	Visual FB (Smartphone, Tablet or PC)	None	N/A	Sensing fabric based on sensor grids; Embedded; normal wire	Body Segment Posture; Improve posture;	Bones trauma or General Rehabilitation
Holden et al. 2015 [71]	Accel (*n* = 2)	Wrist	KR	Concurrent & Terminal; Training notification;	Visual FB (Watch-like device); Vibrotactile FB;	Usab.	Usability test on patients (*n* = 7)	Watch-like device; Attachable;	Amount of Use; Encourage arm hand use;	Stroke
Ongvisatepaiboon et al. 2015; [72]	Accel (Smartphone built-in)	Elbow	KP, KR	Concurrent & Terminal; Shoulder joint angle value, repetition number;	Visual FB (Smartphone)	Tech.	Accuracy test on normal people (*n* = 1)	Armband; Attachable	Body Segment Posture; Improve movement performance;	Frozen shoulder rehabilitation
Lee et al. 2015; [73]	Accel (*n* = 1)	Back	KR	Concurrent; BW FB of unbalance error;	Visual FB (Smartphone); Vibrotactile FB	None	N/A	Based on strips; Embedded, normal wire	Body Segment Posture; Improve posture;	General Rehabilitation
Rahman et al. 2015; [74]	Accel (*n* = 1)	Upper arm	KR	Concurrent; Successful repetition number;	Visual FB (Smartphone Game);	Tech.	Accuracy test on normal people (*n* = 5)	Based on strips; Attachable	Body Segment Posture; Improve movement performance;	Spinal cord injury
Klaassen et al. 2015; [75]	IMU (*n* = 8), KPF strain (*n* = 2), KPF goniometer,	Chest, Upper arm, Forearm, Spine, Hand	KR,KP	Terminal; Range of motion value of body segments; Reaching performance; 3D full body reconstruction;	Visual FB;	Usab.	Usability test on normal people.	Shirt and glove; Integrated; Embedded;	ROM; Improve active joint range of motion;	Stroke
Wang et al. 2016; [76]	IMU (*n* = 3)	Back, Shoulder	KR	Concurrent; Torso and shoulder movement value; BW FB on compensatory movement of torso or shoulder;	Visual FB (Smartphone); Vibrotactile FB; Auditory FB	Tech. Usab.	Accuracy test on normal people (*n* = 7) Usability test on patients (*n* = 8)	Embedded into a wearable garment; Embedded; conductive yarn	Body Segment Posture; Improve posture and movement performance;	Stroke, shoulder pain
Bittel et al. 2016; [77]	Accel (Smartphone built-in)	Arm	KP	Concurrent & Terminal; Angular movement error;	Visual FB (Smartphone)	Tech.	Accuracy and precision test (Comparison experiment with Biodex isokinetic dynamometer)	N/A; Attachable	ROM; Improve active joint range of motion;	General Rehabilitation
Newbold et al. 2016; [78]	IMU (Smartphone built-in)	Low back	KP	Concurrent; Sinification based on stretch forward movement;	Auditory FB	Usab.	Usability test on normal people (*n* = 21);	Strapped with band; Attachable;	ROM; Improve active joint range of motion; Reduce pain;	Chronic pain rehabilitation
Ploderer et al. 2016; [79]	IMU (*n* = 3)	Shoulder, Elbow, Wrist	KP, KR	Terminal; Movement quality, joint ROM value and arm movement heat map; Exercise time and movements number;	Visual FB (PC)	Tech. Usab.	Accuracy test on normal people (*n* = 1); Usability test on therapists (*n* = 8)	Based on Velcro straps; Attachable;	ROM & Body Segment Posture; Improve active joint range of motion; Improve posture and movement performance;	Stroke

*Abbreviations*: *KR* Knowledge of results, *KP* Knowledge of performance, *ROM* Range of motion, *FB* Feedback, *FSR* Force sensitive resistor, *Accel* Accelerometer, *IMU* Inertial measurement unit, *OLE* Optical Linear Encoder, *LDR sensor* Light Dependent Resistor, *BW FB* BandWidth Feedback, *VR* Virtual Reality, *ROM* Range of motion, *Tech.* Technical evaluation, *Usab.* Usability test, *Clini.* Clinical trial, *PC* Personal Computer, *KPF* Knitted Piezoresistive Fabric

### Taxonomy structure

**Fig. 2:**
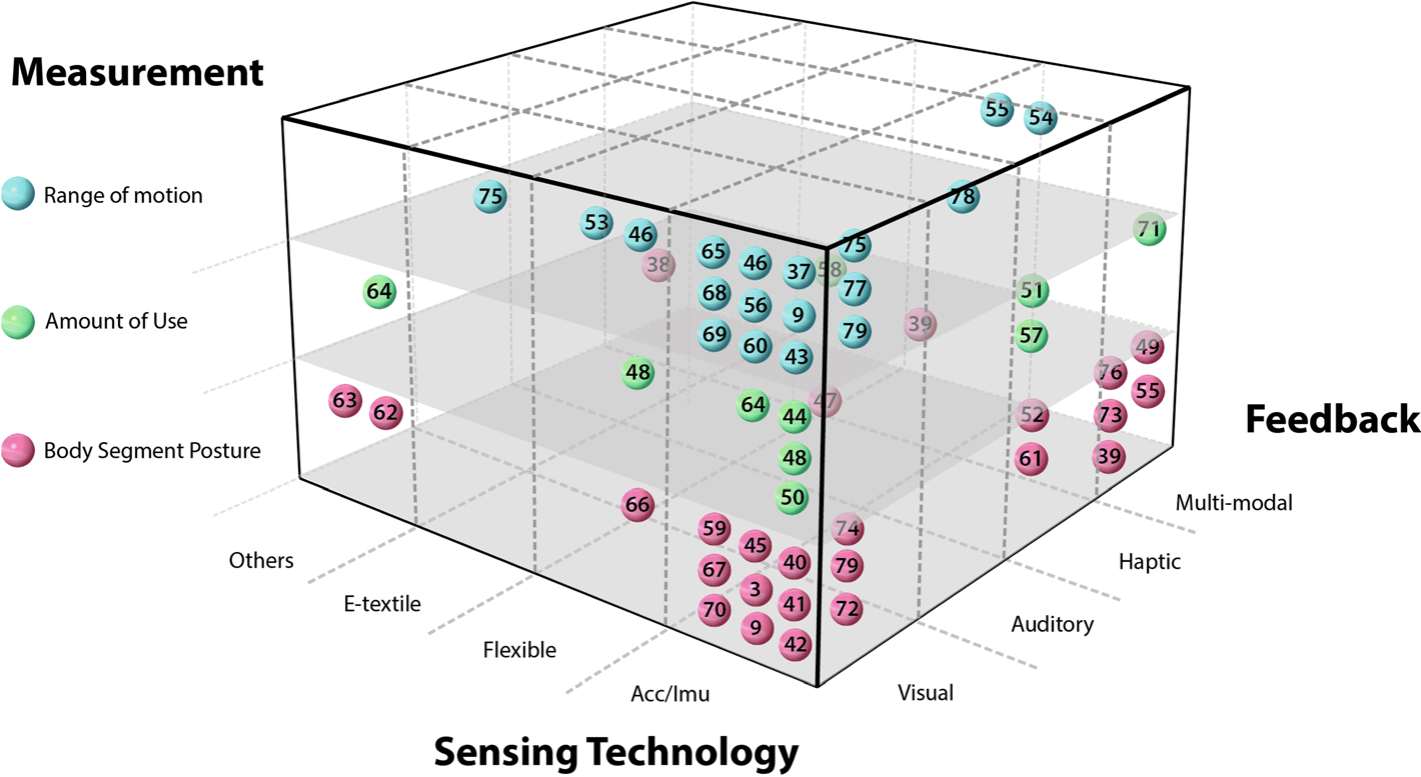
Taxonomy of interactive wearable systems regarding sensing technology, system measurement and feedback modalities

To better understand the emerging phenomenon and classify the systems, a new cuboid taxonomy (shown in Fig. 2) has been proposed, which consists of 3 dimensions: sensing technology, feedback modalities and system measurements. Each dimension pertains to a group of different categories, and has no orientations. These dimensions are key principles for interactive wearable systems for upper body rehabilitation. One dimension is “sensing technology”, it inventories the involved advanced sensing techniques such as Acc/IMU, Flexible angular sensor, E-textile and Others. “Feedback” is another dimension that is essential for interaction between the user and the wearable systems. Feedback concerns different modalities, namely Visual, Auditory, Haptic and Multi-modal modalities. A third dimension is “measurement”. Every system provided different measurements of upper body kinematics which is the basis of building a suitable application for specific pathologies. In our taxonomy, “measurement” includes: Range of Motion, Amount of Use and Body segment Posture. All the 45 articles have been positioned in the cuboid layers, and thereby the features of each system are clearly visualized. Some systems overlap multiple cells. Remarkably, most papers (*n* = 28) are located at the overlap cells of using Accelerometers or IMU sensors and providing visual feedback. We will discuss more details in following sections.

### Status of included sensing technologies

**Fig. 3:**
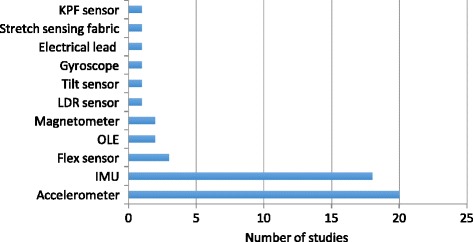
Sensing technology overview. Abbreviations: OLE = Optical linear encoder, IMU = Inertial measurement unit, FSRs = Force sensitive sensor, KPF = Knitted pezoresistive fabric

Figure 3 summarizes the number of studies (horizontal axis) and the different technologies that are used (vertical axis). Some studies involved different technologies in their system. The involved sensing techniques could be classified into 4 categories:

1. Acc/IMU: accelerometer, gyroscope, inertial measurement unit (IMU);
2. Flexible angular sensor: flex sensor, optical linear encoder (OLE);
3. E-textiles: electrical lead, knitted piezoresistive fabric (KPF) sensor, stretch sensing fabric;
4. Others: tilt sensor, magnetometer, light dependent resistor (LDR) sensor.

The accelerometer and IMU sensor are the most frequently used technology within the included feedback systems (used in 38 out of the 45 papers). An accelerometer measures proper acceleration, a gyroscope measures angular velocity, a magnetometer measures magnetic field, and an IMU uses a combination of these three. Systems based on accelerometer or IMU measurements normally consist of several sensor nodes, and can measure kinematic parameters such as orientation, position, velocity, as well as complex body posture and joint range of motion. Micro-electro-mechanical system (MEMS) technology has enabled the development of miniaturized inertial sensors [17].

In 20 studies [3, 37, 40–44, 46, 48–51, 57, 60, 64, 71–74, 77] accelerometer(s) have been integrated: eight of them proposed a single-accelerometer-based system including the studies based on a smartphone built-in sensor, three studies proposed the fusion of an accelerometer with the gyroscope [41], optical linear encoder (OLE) module [46] and flex sensor [48] respectively, while other studies lean on accelerometer combinations.

Eighteen studies [4, 39, 45, 52, 54–56, 59, 61, 65, 67–70, 75, 76, 78, 79] applied IMUs in their systems, three [54, 59, 78] of them relied on a single sensor module. Most systems used 2–4 sensors, but studies that aimed for finger movement monitoring utilized more sensors [68, 69]. Hermanis et al. [70] proposed a novel system that may acquire data from up to 200 sensors, and have demonstrated a smart fabric which integrates 63 sensors in a wearable sensor grid architecture. Two studies [49, 67] used Wii remote as a sensing device and five studies utilized smartphone built-in sensors [54, 59, 72, 77, 78] supporting the growing trend for the use of smartphones for rehabilitation.

A flexible angular sensor includes a flex sensor and OLE strip. Deformation of the substrate of the flex sensor leads to a resistance output correlated to the bend radius. Ambar et al. [48] proposed a multi-sensor system with a flex sensor, force sensitive sensor and accelerometer. OLE consists of an infra-red emitter and a receiver which converts light information in to distance, the infra-red light is reflected off the reflective code strip [46]. Flexible angular sensor arrays have been used on the finger for joint motion tracking. Luo et al. [39] located multi-point OLE strips on different finger segments while Saggio et al. [53] and Halic et al. [66] utilized flex sensors.

Three studies used e-textiles as sensors in their systems. Bhomer et al. [58] proposed a knitted garment based on stretch sensors made of conductive yarn. Klaassen et al. [75] applied “e-textile” goniometers based on knitted piezoresistive fabrics (KPFs), integrated KPF strain and KPF goniometers with IMU’s into a multi-modal sensing system. Friedman et al. [47] located six electrical leads on a glove, registering the electrical connection.

Besides, some researchers explored other metrics. Rahman et al. [62] and Salim et al. [63] proposed a glove-based motion detecting system by integrating LDR sensors and tilt sensors separately.

### System feedback

Feedback is important for rehabilitation training, for supporting the motor learning process in musculoskeletal and neurological pathologies [8, 33], and for sustaining motivation during rehabilitation [7].

#### Feedback modalities

Table 3 classifies the different feedback modalities used in the included studies.

**Table 3:** Systems Feedback

Feedback Modality		Reference
Visual	Abstract (lines, curves, gauges, bars, or point.)	[4, 40, 44, 48, 50, 60, 63–65, 72, 75, 77, 79]
	3D model of limb or human body or structure	[37, 41, 43, 46, 53, 56, 68–70]
	Game	[42, 45, 59, 62, 66, 67]
Haptic	Vibrotactile display	[51, 52, 57, 61]
Auditory	Musical pattern	[78]
Multi-modal		[3, 38, 39, 47, 49, 54, 55, 58, 71, 73, 74, 76]

Visual display is the most common (*n* = 40) way to provide feedback. With visual feedback, the users learn a motor task by therapeutic intervention (training instruction that needs to be achieved) or from the patient him/herself (to compare to the correct/desired movement). In many simple tasks, the task-relevant variable has been represented on a normal screen in a simple abstract form of lines and curves [40, 48, 50, 60, 72, 77], gauges [4, 64], bars [44, 63], or a combination for showing different parameters [65, 75, 79]. For feedback on simple task performance, a numeric or graphic display might be sufficient, since the small number of relevant variables can be meaningfully and directly represented with high information clarity. Besides simple abstract feedback, the global feedback [8] about the posture and position could be provided in a more natural way, which is classified as natural visualizations [84]. The 3D representation could be a virtual teacher/trainer [38, 41, 43, 56] or a 3D model of a limb/hand [37, 53, 56, 68, 69]. To provide quick and accurate feedback, some researches [46] have applied a simplified 3D mechanical model instead of a virtual human model to reduce the rendering time of the image. To motivate the users to practice or train longer, in several systems [42, 45, 59, 62, 67], the visual displays are incorporated into a training game for motor learning, 5 more studies [39, 47, 49, 74] also involved sound or haptic feedback in their games. Besides, some systems combine visual and other modalities as multimodal feedback systems [38, 54, 55, 58, 71, 73, 76] with the aim of enhancing learning effectiveness by reducing the cognitive load required for information processing.

In a study by Fortino et al. [46], a virtual arm was driven by the subject to reach a virtual ball in the simulation environment, while the ball was controlled to move in a predefined route to guide both the real and virtual arm movements. Our results show that virtual reality has been commonly used within the included studies (three studies [37–39] in 2010, two [45, 46] in 2011, one [56] in 2013, three [62, 67, 68] in 2014 and one [69] in 2015). Further to using a computer screen as a visual feedback display, the emergence of smartphones is reflected on the number of the systems providing feedback on smartphones: 0 in 2010–2012, two [54, 58] in 2013, five [59, 60, 63, 64, 66] in 2014, four [70, 72–74] in 2015 and two [76, 77] in 2016.

Vibrotactile displays have been applied in wearable systems for giving information about navigation and directional information [52]. Luster et al. [57], use vibrotactile cues to provide positive reinforcement when performance goals are met during training practice in chronic stroke. The vibrotactile feedback can be located at specific points of interest, such as the forearm [52] or at C7 and T5 level of the spinal column [76], but may also cover a large limb area. Panchanathan et al. [61] developed a flexible vibrotactile strip that can be worn on the body for rich haptic communication. In addition, actuators’ placement for vibrotactile feedback needs to be considered. For example, Ding et al. [38] mentioned the threshold distances for two vibrotactile actuators. These strips may be combined to create wearable two-dimensional haptic feedback. The capability of haptic feedback for presenting precise or complex information is limited, therefore they are often used in combination with visual/ audio feedback as a multimodal feedback [38, 71, 73, 76].

Although only one study utilized auditory feedback as the exclusive feedback modality in their system [78], Newbold et al. [78] explored musically-informed movement sonification for stretching exercises, using stable sound to facilitate stretching exercises and unstable sound to avoid overdoing. Auditory feedback plays important role within the studies providing multi-modal feedback. For example, as a simple and clear notification of error or reward, e.g., as a beeping sound [76]. Furthermore, Bhomer et al. [58] proposed a more complex system in which the sound reflects the movement of the wearer as the pitch or volume of a tune is controlled by the stretch of a fabric sensor. Friedman et al. [47] encouraged the subject to hit notes with music feedback to practice hand function.

#### Feedback content and timing

Regarding to the content of feedback, most wearable systems present the skill outcome or goal achievement, defined as knowledge of results (KR) [83]. Examples are the summary feedback of the achieved number of specific training activities [44], movement parameter scores (range of motion, quality of movement) [4], successful repetition number [45, 50, 72, 79]. Knowledge of performance informs about the movement characteristics that led to the performance outcome [83]. One common way is to present kinematic information such as position, time, velocity, and patterns [37, 41, 45, 46, 60]. Ding et al. [52] and Panchanathan et al. [61] proposed feedback on arm movement performance by vibrotactile feedback on directing towards the correct posture. Panchanathan et al. [61] also indicated the speed errors and how to correct them. Within the included studies, 16 studies applied KR feedback, 14 studies applied KP feedback and 16 studies applied both.

Eleven studies utilized game scenes to make repetitive movement more engaging for the patient and to motivate them to practice or train longer. Examples are grasping activities [39, 67], arm or finger movement performance [47, 59, 62, 66, 74], upper limb trajectory indication [45], and feedback based on compensatory movements within the games [3, 42, 49].

Bandwidth feedback is defined as feedback given only when a movement error exceeds a certain threshold [84]. Bandwidth feedback is beneficial for personalized feedback to individual patients. Four papers [3, 42, 49] set compensatory movement limits as the trigger for game effects; another three studies used the reference position as a threshold [52, 76, 79].

With regard to timing, feedback can be given during the training execution (concurrent feedback) or after completion of the training (terminal feedback) [84]. Concurrent feedback has been suggested to be effective for beginning users and terminal feedback may benefit more the skilled user [8]. Most included studies (*n* = 29) applied concurrent feedback strategies, 11 studies used both concurrent and terminal feedback, only 5 studies used terminal feedback, 4 of them by means of KR feedback and one study applied both.

### Measurement

Wearable systems for the registration of body segment joint kinematics, give feedback on movements like flexion, extension, abduction, adduction, rotation and parameters such as time and speed. Hence the dimension “measurement” could be classified into: range of motion (movement distance around joint or body part), amount of Use (activity amount of body segment) and body segment posture (specific posture or body segment to target spatial location). Similar measurements may support various rehabilitation purposes and patient populations. Details of each study are presented in Table 2.

#### Measurement for different rehabilitation purposes

The included studies for upper body rehabilitation, had following aims: improve active joint range of motion, improve movement performance, improve movement coordination, improve posture, improve muscle strength, overcome learned non-use and improve performance of ADL (activities of daily living) skills.

Sixteen studies [4, 37, 43, 46, 53–56, 60, 65, 68, 69, 75, 77–79] focused on the measurement of range of motion (ROM) with the common purpose of improving active joint range of motion. Studies by Timmermans et al. [4] and Parker et al. [65] also concentrated on improving ADL skills for Stroke. Harms et al. [53] aimed at improving posture and Newbold et al. [78] aimed at reducing pain during rehabilitation in chronic pain patients.

The “Amount of Use” is used in 8 studies [44, 48, 50, 51, 57, 58, 64, 71]. Two studies [44, 57] targeted at bilateral arm movement detection (use) to overcome learned non-use and 2 studies [44, 48] mentioned improving ADL skills. Jeong et al. [50], Myllymaa et al. [51], Bhomer et al. [58], Friedman et al. [64], and Holden et al. [71] intended to motivate the amount of exercise during general rehabilitation.

The category “Body Segment Posture” include 24 studies [3, 4, 38, 39, 40–42, 45, 47, 49, 52, 55, 59, 61–63, 66, 67, 70, 72–74, 76, 79] about measurement of specific posture such as compensatory movement [80] and motion guidance. Most (16 out of 24) systems aimed for improving movement performance as these studies help users understand the desired motions and guide them through correct movement patterns, followed by 7 studies for improving posture, two for improving ADL [3, 47] skills and one for improving coordination [47].

#### Measurement for different target population

In addition, we inventoried the target population addressed by interactive wearable systems (Table 4). Three categories are identified: 1) Neuro-rehabilitation: stroke (*n* = 21), spinal cord injury (*n* = 1), cerebral palsy (*n* = 2), Alzheimer (*n* = 1); 2) Musculoskeletal impairment: ligament rehabilitation (*n* = 1), arthritis (*n* = 1), frozen shoulder (*n* = 1), bones trauma (*n* = 1); 3) Others: chronic pulmonary obstructive disease (*n* = 1), chronic pain rehabilitation (*n* = 1) and other general rehabilitation (*n* = 14).

**Table 4:** Classification based on target population

Target Population		Reference
Neuro-Rehabilitation	Stroke	[3, 4, 38, 39, 44–49, 52, 53, 57, 59, 63, 65, 67, 71, 75, 76, 79]
	Spinal cord injury	[74]
	Cerebral palsy	[42, 62]
	Alzheimer	[58]
Musculoskeletal impairment	Ligament rehabilitation	[40]
	Arthritis rehabilitation	[69]
	Frozen shoulder rehabilitation	[72]
	Bones trauma	[70]
Others	COPD (chronic obstructive pulmonary disease)	[54]
	Chronic pain rehabilitation	[78]
	General rehabilitation (hand, elbow, shoulder, total upper extremity), no specific pathology	[37, 41, 43, 50, 51, 55, 56, 60, 61, 64, 66, 68, 73, 77]

### System wearability

#### Sensor placements

**Fig. 4:**
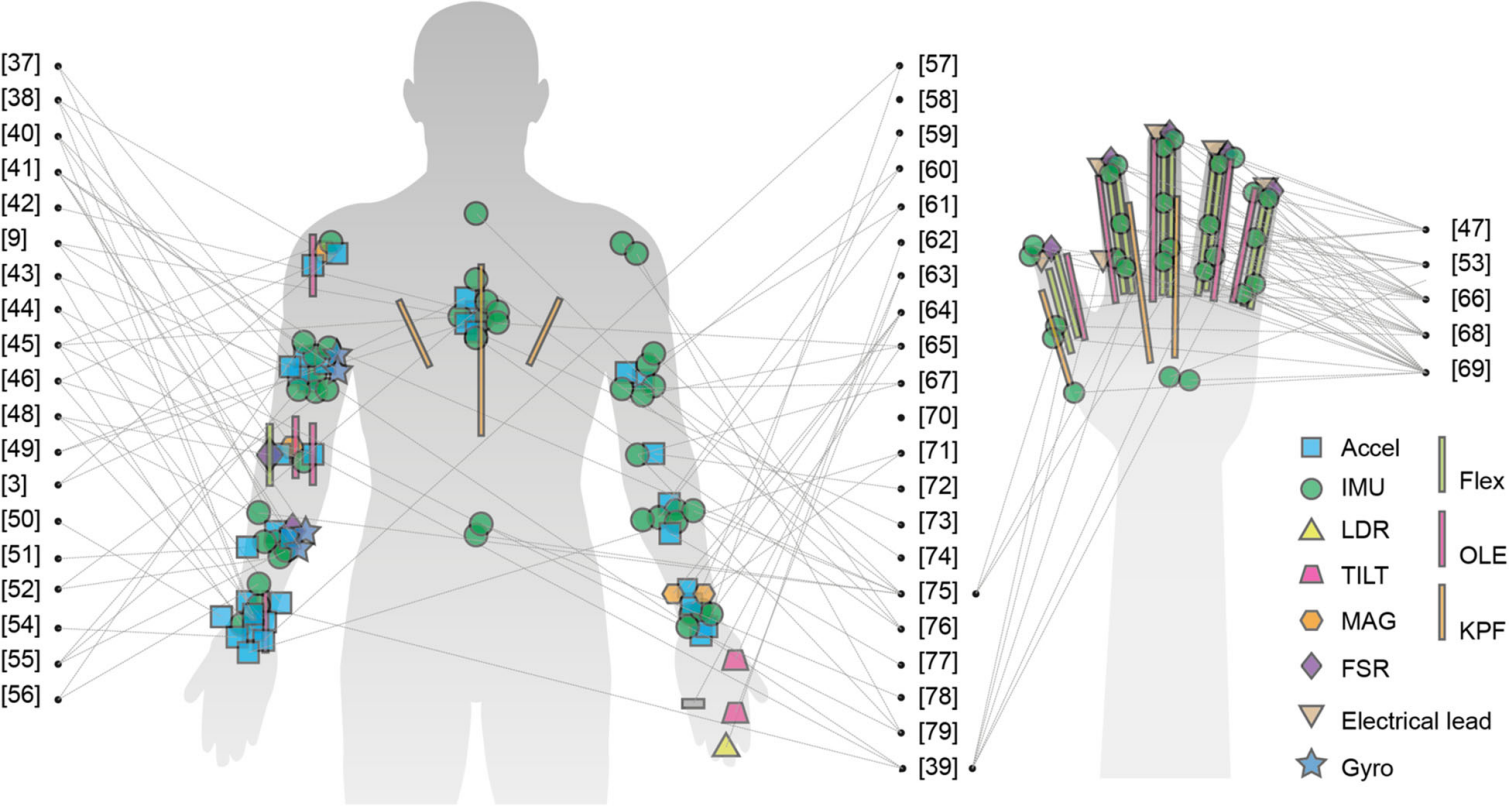
Infographic of sensor placements

Figure 4 illustrates the sensor placement for all the studies included in this review with the intention of showing an overview of the sensing module distribution on the upper body. The papers of Hermanis et al. [70] and Bhomer et al. [58] have not been included in this figure, since the sensor grid system [70] is capable of acquiring up to 63 sensors as a smart surface that can be worn on the back in the form of a blazer vest and the sensing areas knitted garment [58] based on smart textiles could cover the upper body instead of specific points. For the remaining articles, we have found that the main concentration of sensors is on upper arm (*n* = 16), forearm (*n* = 11), wrist (*n* = 14), elbow (*n* = 9), trunk (*n* = 13 including location on chest and back) and finger (*n* = 7).

#### Wearable design

Wearability has been defined by Gemperle et al. [81] as the interaction between the human body and wearable objects. Wearability is one of the key aspects for the acceptance of wearable systems; especially wearable systems that are aimed for long-term monitoring have high requirements for comfort.

From a system implementation perspective, the integration level of electronics and textile influences the wearability to a high extent. The integration level pertains to how electronic parts are embedded in a wearable system. Based on Seymou et al. [82], the integration level is distinguished into following categories: 1) Attachable, using a container like pocket or strapped with bands; 2) Embedded, sensing parts physically embedded into fabric, such as by conductive yarns; 3) Integrated, smart textiles sensors. In the second category, there are two ways to embed the sensing parts into the wearable system: with standard copper wires and with conductive yarns. Various ways of locating the sensors in the right places have been proposed. To be more specific, this review classified as follows: a) most included systems are in the stage of being *attachable* (*n* = 29) [3, 4, 37, 39–42, 44, 45, 48–55, 59, 60, 64–67, 71, 72, 74, 77–79], which is easy for prototyping and easy for operation of the system with a single device [42]; b) fewer studies are in the stage of *embedded systems with normal wires* (*n* = 10) [38, 43, 46, 56, 57, 62, 63, 68, 70, 73]; c) for even fewer systems sensors are *embedded in the fabric with conductive yarns* (*n* = 2) [61, 76]; d) integrated into *smart textiles* (*n* = 3) [47, 58, 75]. Besides, O’flynn et al. [69] proposed a glove combined stretchable substrate material and IMUs by customized PCB board doesn’t require fabric platform.

**Fig. 5:**
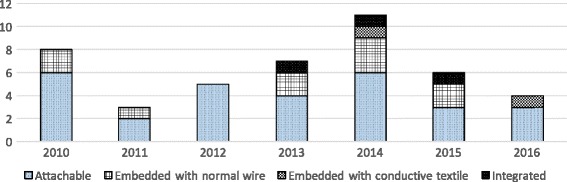
Overview of Integration classification

Figure 5 summarizes the number of studies in different type of integration and in different years. Compared to systems in attachable level, embedded systems are more aesthetic and less bulky. Although the systems in integrated level with fabric-based sensing enhanced both comfort and aesthetics, the accuracy and flexibility supporting multi-DOF is limited [58, 69]. However with the emerging developments in smart textiles [32, 75], fabric-based sensing are showing great potential.

#### Wearable factors and requirements

**Fig. 6:**
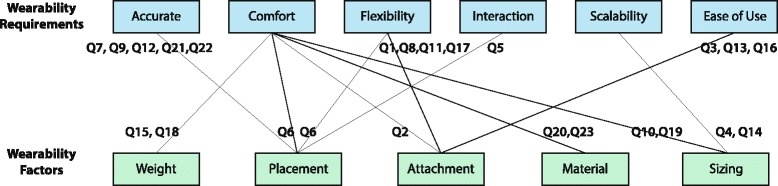
Wearability factors supporting wearable system requirements

Apart from system implementation issues, the efforts on improving the systems wearability can be classified in three levels: proposing a sensor package/platform design criteria/requirements [44, 47, 55, 56, 76]; including wearability related questions during the evaluation of the system with users [57] and, finally, reporting lessons learned about system wearability [45, 50, 61]. Table 5 summarizes claims made about wearability in these articles. Although the wearable systems are quite different, these quotes demonstrate current design requirements for wearability and how factors pertaining to wearability support these requirements. The relationship is illustrated in Fig. 6.

**Table 5:** Quotes list about wearability requirements from included studies

Ref	Quotes from included studies
[46]	Q1. “does not restrain the human movement”; Q2. “without slipping on users’ skin”; Q3. “be easy to wear”; Q4. “fit to human arms with different size”;
[52]	Q5. “consideration of minimum critical distance for two adjacent vibrotactile actuators”
[55]	Q6. “unobtrusive and not limit the skin and muscle motion”; Q7.”place sensor on bones, ligaments and between muscles”; Q8. “with some flexibility in positioning”; Q9. “provide additional stability”;
[56]	Q10. “must be non-invasive to be accepted by patient”; Q11. “have to avoid restraining the movements that the patient does in normal conditions”;
[76]	Q12. “fit closely to body for higher accuracy”; Q13. “Easy to wear on and off”; Q14. “adjustable for different size”; Q15.”light, comfortable, appropriate for long term monitoring”;
[57]	Q16. “how easy to put on/ take off the suit”; Q17. “how easy was it to move your affected arm compared to without wearing the wristband”; Q18. “how comfortable/lightweight were the wristbands”;
[44]	Q19. “module size was too large, draw attention”
[61]	Q20. “reduce the quantity and bulk of the wiring”
[49]	Q21. “attach the harness around the neck, not the shoulders”; Q22. “stabilize the Wii Remote against the back to prevent rolling”; Q23.”with a soft cloth cover to prevent rubbing against the skin”;

Based on Table 5 and Fig. 6, following aspects has been concluded:

1. Accuracy: the wearable should help locate and keep the sensor in the right location on the body for high accuracy (Q7,9,10,19,20).
2. Comfort: wearable factors contribute both physiological comfort and psychological comfort (Q10,19); the system should be light (Q15,18), unobtrusive with suitable material (Q20,23) and attachment methods (Q2).
3. Flexible: the system should guarantee human movement flexibility (Q1,6,8,11,17).
4. Interactive: the wearable systems should support interactive therapy (Q5);
5. Scalable: the system should address body size diversity (Q4,14);
6. Ease of use: the system should be easy to operate and easy to put on and take off (Q3,13,16).

### Evaluation

**Fig. 7:**
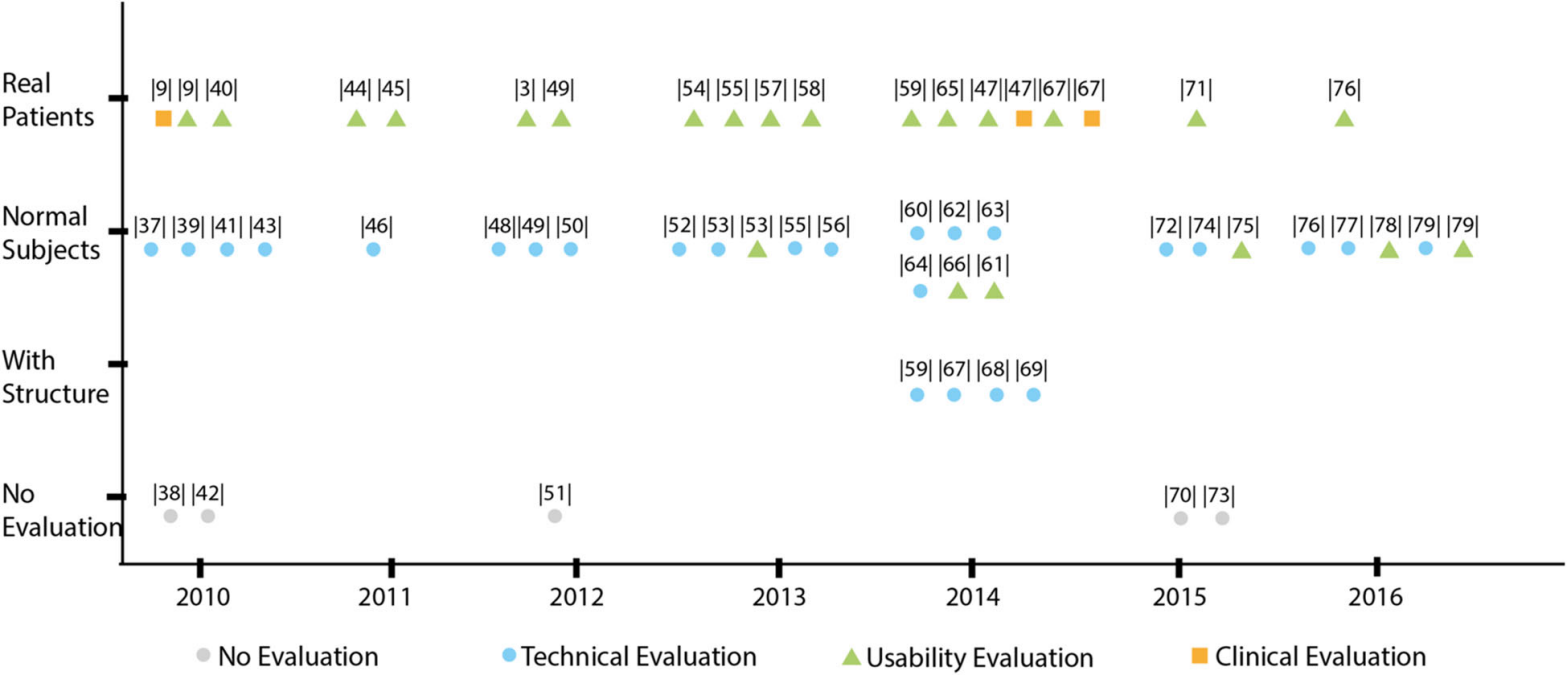
Systems evaluation

The included systems are classified into four stages based on their evaluation status: a) no evaluation (*n* = 5); b) technical evaluation (*n* = 25); c) clinical trials (*n* = 3); d) usability test (*n* = 17), while six systems [49, 53, 55, 59, 76, 79] conducted both technical and usability evaluation in their studies and one study [67] conducted all. Some studies report evaluations from different perspectives. It is noteworthy that not all the experiments described in the studies could be defined as evaluation evidence. There are five studies that didn’t provide evaluation evidence. Note that the availability of “evaluation evidence” was not used as an inclusion criterion in this study, in order to not exclude reports on very novel systems that are presented as proof of concept/principle as, for example, the smart fabric embedded wearable sensor grid discussed above [70]. Figure 7 Illustrates the systems evaluation status in details.

The technical evaluation was conducted with regard to the following aspects: accuracy, sensitivity, reliability, power consumption and feasibility. There are 25 studies that describe a technical evaluation along such requirements, 21 studies didn’t include patients and conducted the experiment only with healthy subjects.

Most sample sizes in the empirical evaluation studies reported are relatively small, ranging from 1 to 10, while only seven studies [47, 48, 50, 55, 61, 66, 78] involved more than 10 subjects, Halic et al. [66] conducted a usability evaluation with 46 subjects.

Although 16 of the included studies involved patients and reported usability tests, only three of these were clinical trials [4, 47, 67] including one randomized clinical trial [47] with 12 chronic stroke survivors for 2 weeks. From Fig. 7, we can see that the usability evaluation with patients is drawing more attention from 2010 to 2014.

## Discussion

This paper reviews the featured technologies developed over the recent 6 years, focusing on interactive systems of wearable-sensing based technology toward upper body rehabilitation. We proposed a taxonomy that consists of 3 dimensions: measurement, sensing technology and feedback. This new taxonomy may benefit other researchers to gain deeper understanding of the emerging projects, have more insights and explore the promising design space.

### Discussion of wearable-sensing technologies

Advanced technologies have been developed and applied to solve the relevant application problems [27]. Various electronic sensors and systems have been applied in these studies, namely: accelerometer, gyroscope, inertial measurement unit (IMU), flex sensor, optical linear encoder (OLE), magnetometer, force sensitive sensor (FSRs), light dependent resistor (LDR sensor), tilt sensor, electrical lead, knitted piezoresistive fabric sensor (KPF) and stretch sensing fabric. The accelerometers and IMU’s tend to be the most commonly used with the following advantages: they yield accurate essential values, are easy to use and are miniature in size.

Some new developments on innovative sensing technologies are noteworthy and promising though they have been excluded from the survey as they are only sensing technologies which do not support yet any user feedback: conductive thread based stretch sensors [85], a conductive elastomer sensor based system [20], stretchable carbon nanotube strain sensors [86] and soft nano-patches [87]. Based on the review study by Fleury et al. [32], the development of conductive elastomer sensors have primarily affected the recent advancements of textile-based motion sensing, providing comfortable garments with high integration level of electronic components and fabric. Although conductive elastomer based systems show accurate performance compared to IMU sensors, the single axis measurement and languid response limits their application for rehabilitation.

Besides, the sensing placement plays an important role for upper body rehabilitation as a combination of locations can provide the value of range of motion (ROM) assessment, body segment position, usage and position. These values are crucial for rehabilitation therapy as their observation and interpretation influence how the treatment develops [88].

### Discussion of systems feedback

It is important that feedback matches the proficiency level of the users [8]. The majority of systems (*n* = 29) included in this review use concurrent feedback which is mostly suitable for persons that are not proficient. Only 5 systems use terminal feedback and 4 of them by means of knowledge of results. There is a lack of systems that use fading frequency schedules that match the frequency of feedback provision to the progress of the patient: the more proficient the user, the less frequently feedback needs to be given so persons don’t get dependent on the extrinsic feedback and learn to rely on their intrinsic feedback mechanisms [8]. This is a point of attention for future system developments.

Several feedback modalities were used. The natural visualization displays the movement of the user’s body simultaneously with a virtual 3D modal. It could enhance the user’s learning by imitation [91]. Also users may enhance motor learning by mental practice, where similar brain areas are active than during overt motor actions [92].

Haptic and audio feedback do not require visual attention during the exercise. Haptic feedback, especially vibrotactile displays, are widely used (*n* = 8) in the systems included in the study. Haptic feedback allows patients to focus on specific body areas rather than divide their attention to a visual or auditory display. Vibrotactile feedback has been used to notify users on joint angle related errors and on speed of movement [61]. Vibrotactile feedback is also capable of presenting KP feedback [51, 52]. Auditory feedback as a substantial modality has been applied as an exclusive feedback by one study, Newbold et al. [78] explored musically-informed movement sonification. Bhomer et al. [58] and Friedman et al. [47] proposed systems in which the sound together with screen feedback reflects the movement of the wearer. Other studies applied auditory alarms as bandwidth feedback when a certain movement exceeds the threshold as an error notification [3, 60] or as notification for rewards [49].

Virtual reality technology has been used extensively in the included studies. Considering the recent booming development of VR technology and serious games, these technologies offer enormous potential for increasing the training intensity, engagement and social participation for patients.

Recent advances in smartphone technology such as their prevalence, ability to use anywhere, powerful processing ability and integration of sensor and display have had a major impact on their use in rehabilitation systems. Providing feedback like visual information on smartphones is common and effective, especially for the systems intended for remote monitoring.

### Discussion of system wearability

Most articles have conducted a technical, a usability, and more rarely a clinical evaluation (only 3), while none of the included studies report a systematic wearability assessment, which is quite essential for user acceptance. Most included studies describe only superficially how to attach sensors on the human body, despite that the way this placement is done is very influential on both accuracy and comfort of the system.

Regarding the different sensing technologies and four integration levels of electronics and textiles, most studies in category Acc/IMU are restricted to the lowest level of integration where devices are attached to the body rather than integrated in a wearable system through ad hoc contraptions (e.g. Velcro strips), and sensors are distributed on body segments (e.g. upper arm, forearm and wrist) to work as a combination system. However the studies within embedment level are increasing and have the advantages of stability, comfort, unobtrusiveness and feasibility. Studies in the category “Flexible angular sensor” are embedded sensors in a suitable platform and precisely located at body joint (e.g. elbow). Two studies in category “Others” embedded the sensor in gloves. Only three studies are in the integrated level based on smart textiles. However, applying smart textiles for posture detection, such as resistance changing materials, pressure-sensitive conductive sheets, knitted conductive textile and conductive yarns are growing trends in the area of wearable electronics that should soon be reflected in the domain of wearable rehabilitation technology [32, 89]. Currently, considering the rehabilitation context, “Acc/IMU” show superiority for projects with a high requirement of kinematic accuracy, while for a high preference of user experience the category “E-textile” has more advantages.

The reviewed studies have identified a number of requirements that may be key to improve wearability and usability of wearable rehabilitation technology: accuracy, comfort, interactiveness, flexibility, scalability, and ease of use. There has been little effort yet to evaluate wearability. In this respect, the study by Cancela et al. [90] is an inspiring example, where the Comfort Rating Scale was used to assess perceived exertion and physiological and biomechanical parameters were assessed to measure musculoskeletal loading.

### Discussion of clinical validation

Only 3 systems have been clinically evaluated in clinical pilot trials [4, 67] and one randomized clinical trial [47] has been found. Compared to the results of the review study by Timmermans et al. [8], there have been only small improvements of the clinical evidence on wearable sensor-based systems. This can be attributed to the long time that technological developments require, and the fact that premature systems do not justify the time consuming and costly process of (randomized) clinical trials.

Twenty-one out of the 45 studies aim for stroke rehabilitation. The focus on stroke rehabilitation is in line with the general developments in the field of rehabilitation technology. However, it is surprising with regard to developments in wearable sensor systems for rehabilitation as they are mostly targeting a combination of posture monitoring in combination with upper extremity movement monitoring which is of great value for musculoskeletal as well as neurological pathologies. Compared to other wearable systems that support clinical applications [12] for lower extremity rehabilitation and physical activity recognition, the clinical validation proportion of wearable-sensing systems for movement measurement during upper body rehabilitation shows disparity.

Clinical trials are important to assess the effectiveness of the systems with regard to the additional clinical value they may provide to the patients for improving their condition. Such trials are also paramount to pave the path towards implementation in clinical settings, as therapists will be hesitant to use them without clinical validation studies [93].

### Inspirations from novel wearable concepts

Researchers working on wearables from the field of textile and fashion design and from the field of human computer interaction have been developing inspiring wearable solutions; although their objectives may not focus on rehabilitation, their work shows the future trend that can enhance wearable systems for rehabilitation:

1. Textile displays as visual feedback. For example, textile display based on thermo paint [94]. Based on the sensitive property of the thermo paint, both concurrent feedback and long-term feedback (e.g. after one hour’s training) could be provided. Or display technologies such as embedded mini LEDs or optical fibers can be embedded into clothing.
2. New forms of haptic feedback, such as inflatable interfaces like the dynamic textile forms (e.g. origami textile structure [95]) that move.
3. Personalized design and digital fabrication, adapting their form and functionality based on individual needs can be realized through 3D scanning and 3D printing techniques [96]. Customization design opens the opportunity of accurately and comfortable locating the sensors for individual patients.

## Conclusions

Researchers from different backgrounds in biomedical science, engineering, computer science, and rehabilitation sciences have cooperated towards the development and evaluation of wearable systems for upper body rehabilitation. The results indicated that accelerometers and IMUs were most commonly used and they were used to monitor and provide feedback to patients on range of motion and movement performance during upper body rehabilitation. New possibilities are arising with up-coming technologies such as e-textiles and nano-sensors. Most systems were in the stage of feasibility prototypes, where only technical evaluations have been conducted. Some systems have reached the maturity to support user tests, while only three systems have been evaluated in clinical trials. There is a growing trend for using the smartphone as a monitoring device and as a feedback carrier. Rehabilitation training may be further improved when wearable sensing hardware takes enhanced wearability into account. Future research should focus on integrating advanced textile sensors, improving usability, wearability as well as clinical validation. The latter is of high importance to pave the path towards implementation into clinical practice.

## Abbreviations

Accel: Accelerometer
BW FB: Bandwidth Feedback
Clini.: Clinical trial
FB: Feedback
FSRs: Force sensitive sensor
IMU: Inertial measurement unit
KP: Knowledge of performance
KR: Knowledge of results
LDR sensor: Light Dependent Resistor
OLE: Optical Linear Encoder
PC: Personal Computer
ROM: Range of motion
Tech.: Technical evaluation
Usab.: Usability test
VR: Virtual Reality
